# Harnessing the immune system to overcome cytokine storm and reduce viral load in COVID-19: a review of the phases of illness and therapeutic agents

**DOI:** 10.1186/s12985-020-01415-w

**Published:** 2020-10-15

**Authors:** Sumanth Khadke, Nayla Ahmed, Nausheen Ahmed, Ryan Ratts, Shine Raju, Molly Gallogly, Marcos de Lima, Muhammad Rizwan Sohail

**Affiliations:** 1 Our Lady of Fatima University, 120 MacArthur Highway, 1440 Valenzuela, Metro Manila Philippines; 2 Section of Hospital Medicine, Dartmouth-Hitchcock Medical Center - Geisel School of Medicine, One Medical Center Drive, Lebanon, NH 03766 USA; 3 Section of Hematology Oncology, Bone Marrow Transplant and Cellular Therapy, University Of Kansas Medical Center, 3901 Rainbow Blvd, Kansas City, KS 66160 USA; 4 Section of Pediatric Hospital Medicine, Dartmouth-Hitchcock Medical Center - Geisel School of Medicine, One Medical Center Drive, Lebanon, NH 03766 USA; 5 Section of Pulmonary and Critical Care, University Hospitals Cleveland Medical Center - Case Western Reserve University, 11100 Euclid Avenue, Cleveland, OH 44106 USA; 6 Section of Hematology Oncology, Stem Cell Transplant and Cellular Therapeutics, University Hospitals Seidman Cancer Center - Case Western Reserve University, 11100 Euclid Avenue, Cleveland, OH 44106 USA; 7 Section of Infectious Diseases and Cardiovascular Medicine, Mayo Clinic College of Medicine and Science, 200 1st St SW, Rochester, MN 55905 USA

**Keywords:** COVID-19, SARS-CoV-2, Pathophysiology, Cytokine release syndrome, Angiotensin converting enzyme 2, Acute respiratory distress syndrome, Tocilizumab, Immunotherapy, Antiviral, Chloroquine

## Abstract

**Background:**

Coronavirus disease 2019 (COVID-19) is caused by Severe Acute Respiratory Syndrome Coronavirus 2 (SARS-CoV-2, previously named 2019-nCov), a novel coronavirus that emerged in China in December 2019 and was declared a global pandemic by World Health Organization by March 11th, 2020. Severe manifestations of COVID-19 are caused by a combination of direct tissue injury by viral replication and associated cytokine storm resulting in progressive organ damage.

**Discussion:**

We reviewed published literature between January 1st, 2000 and June 30th, 2020, excluding articles focusing on pediatric or obstetric population, with a focus on virus-host interactions and immunological mechanisms responsible for virus associated cytokine release syndrome (CRS). COVID-19 illness encompasses three main phases. In phase 1, SARS-CoV-2 binds with angiotensin converting enzyme (ACE)2 receptor on alveolar macrophages and epithelial cells, triggering toll like receptor (TLR) mediated nuclear factor kappa-light-chain-enhancer of activated B cells (NF-ƙB) signaling. It effectively blunts an early (IFN) response allowing unchecked viral replication. Phase 2 is characterized by hypoxia and innate immunity mediated pneumocyte damage as well as capillary leak. Some patients further progress to phase 3 characterized by cytokine storm with worsening respiratory symptoms, persistent fever, and hemodynamic instability. Important cytokines involved in this phase are interleukin (IL)-6, IL-1β, and tumor necrosis factor (TNF)-α. This is typically followed by a recovery phase with production of antibodies against the virus. We summarize published data regarding virus-host interactions, key immunological mechanisms responsible for virus-associated CRS, and potential opportunities for therapeutic interventions.

**Conclusion:**

Evidence regarding SARS-CoV-2 epidemiology and pathogenesis is rapidly evolving. A better understanding of the pathophysiology and immune system dysregulation associated with CRS and acute respiratory distress syndrome in severe COVID-19 is imperative to identify novel drug targets and other therapeutic interventions.

## Introduction

Since it was first reported from Wuhan, China in December 2019, Coronavirus Disease 2019 (COVID-19) has rapidly spread across the globe and was declared a global pandemic by the WHO on March 11th, 2020 [1]. As of July 18th, 2020, 188 countries have been affected with more than 14 million confirmed cases and over 600,000 fatalities [2]. Being a novel virus, there has been a steep learning curve about its microbiology, host interactions, mechanism of immune dysregulation in humans, and tissue injury. Multi-modality therapeutic options are being explored on an emergent basis with limited evidence of efficacy [3].

We provide a focused review of the published literature regarding the pathophysiology of COVID-19 with an emphasis on the anti-viral and immunomodulatory therapies.

## Methodology

We conducted searches on PubMed and Google Scholar for any articles between January 1st, 2000 and June 30th, 2020, with the search terms “Coronavirus or COVID-19” in conjunction with the search terms “transmission”, “pathogenesis”, “immune response”, “cytokines”, “interleukin (IL) inhibitor”, “antiviral therapy”. Due to limited published literature related to COVID-19 in the pediatric and obstetric population and their unique aspects, we exclude articles pertaining to that population. We also reviewed information published on the World Health Organization (WHO), Centers for Disease Control and Prevention (CDC), and John Hopkins University Center for Systems Science and Engineering (CSSE) websites.

## Epidemiology

The Huanan Seafood Wholesale Market in Wuhan, China, the purported origin site of Severe Acute Respiratory Syndrome Coronavirus 2 (SARS-CoV-2), was the epicenter of new cases of COVID-19 from December 2019 to January 2020. In February 2020, the epicenter shifted initially to Italy and Spain, and subsequently to the United States of America (USA) in March 2020 [4, 5]. Estimated case fatality rate with COVID-19 ranges from 0.5 to 3% [6, 7]. However, mortality is higher in males, patients with comorbidities including diabetes mellitus, heart disease or hypertension, and those over age 60 years [8, 9].

Wu et al. reviewed the epidemiology of 72,314 COVID-19 patients in China and noted that the predominant age distribution was 30–79 years of age, but observed increasing case fatality rate (CFR) in older patients (> 80) [10]. Less than 2% of identified patients were less than 18 years of age. While similar patterns have been reported in Europe and the United States, the interpretation of epidemiological data is limited by the testing characteristics of the specific community, with likely under-representation of asymptomatic patients [2]. Further, variable transmission rates are also observed based upon characteristics of the local community (e.g. urban vs rural, age distribution, etc*.*) and any public health policies in place for containment or mitigation such as quarantines, shelter-in-place orders, mask-wearing, or contact tracing.

## Clinical presentation

The spectrum of clinical manifestations ranges from asymptomatic to life-threatening. However, more than 80% of patients have mild symptoms or are asymptomatic [11, 12]. The most frequently reported symptoms include fever (80–90%) and dry cough (50–70%). There may be associated severe fatigue and dyspnea. Loss of taste and smell have also been reported. Gastrointestinal symptoms (nausea, vomiting, diarrhea) are present in less than 5% of patients. Symptoms typically resolve within 5–10 days. However, approximately 14% of patients have severe disease requiring hospitalization and 5% may have critical illness evidenced by adult respiratory distress syndrome (ARDS), respiratory failure, shock and/or multi-organ dysfunction [13, 14].

## Predictors of severe disease

Clinical predictors of poor outcome include advanced age, male gender, hypertension, diabetes mellitus and coronary artery disease [15, 16]. Laboratory predictors of critical disease include lymphopenia, elevated levels of D-Dimer, pro Brain-type Natriuretic Peptide (pro-BNP), troponin I, and creatinine [9, 15, 16]. High levels of inflammatory markers such as IL-6, C-reactive protein (CRP), and ferritin are also associated with more severe disease [17]. Qin et al. described that patients with severe COVID-19 infection had significantly lower circulating B cells, T cells, and Natural killer (NK) cells on flow cytometry as compared to non-severe cases, endorsing the hypothesis that immune dysregulation plays a role in disease severity [18].

## Mode of transmission

SARS-CoV-2 is a member of the betacoronavirus (β-CoV) family. In the last 20 years, the most lethal strains of the β-CoVs causing epidemics include Severe Acute Respiratory Syndrome Coronavirus (SARS-CoV) in 2002, Middle Eastern Respiratory Disease coronavirus (MERS-CoV) in 2011 and SARS-CoV-2 in 2020. SARS-CoV-2 is purportedly spread to humans from bats via intermediate hosts such as turtles and pangolins, however this is currently controversial [19–23]. The main mechanism of spread for both SARS-CoV and SARS-CoV-2 seems to be human-to-human transmission [3, 20]. COVID-19 is predominantly thought to be spread via droplets and fomites [24], with very limited aerosolization, and recent data indicates possible fecal–oral spread as well [11, 24, 25]. Patients can be contagious for 24–48 h before symptom onset [6, 10]. The incubation period is 2–15 days, with a mean of 5.1 days. Most (97.5%) patients develop symptoms within 11.5 days [11]. The virus can survive up to 1–2 days on glass and metal surfaces, and up to 4–5 days on plastic surfaces [26]. It is unclear if a significant amount of SARS-CoV-2 is present in breast milk, urine, or semen for transmission. Vertical transmission from pregnant mothers to infants remains a controversial topic, but there are emerging reports of damage to the placenta from COVID-19 [24], and SARS-CoV-2 RNA has been detected on the fetal side of the placenta [27].

## Testing

Microbiologic diagnosis of SARS-CoV-2 is made by real-time reverse transcriptase-polymerase chain reaction (rt RT-PCR), serology, and rapid antigen detecting kits [28]. True sensitivity of PCRs from nasopharyngeal swabs varies from 30 to 70% depending on the phase of illness [29]. Since PCR has a significant false-negative rate, a negative PCR should be interpreted in context with the clinical manifestation, disease phase, and radiological findings. Virus-specific immunoglobulins (Ig)—IgG and IgM antibodies can be detected beyond day 5 of infection and can be detected in those who have active disease or recovered, although delays in seroconversion beyond 14 days have been reported [30]. Further, there are some reports that antibodies produced against SARS-CoV-2 are short-lived and may not be fully protective [31]. The sensitivity and specificity of serologic assays differ based on the specific methodology utilized (e.g. ELISA, agglutination, or complement-fixation). Numerous serology kits with variable false negative and false positive rates are currently in the process of being developed, and appropriate implementation requires validation.

Serological testing as a diagnostic tool for COVID-19 is limited by the fact that seroconversion may be significantly delayed after the onset of illness, although it may have increasing utility during later phases of disease when viral loads are lower [32]. A clearer understanding of the kinetics of antibody production during infection is critical for understanding the specific role of serological testing as a diagnostic tool, as well as an instrument for seroepidemiological and vaccine evaluation studies [33].

## SARS-COV-2 structure

**Fig. 1:**
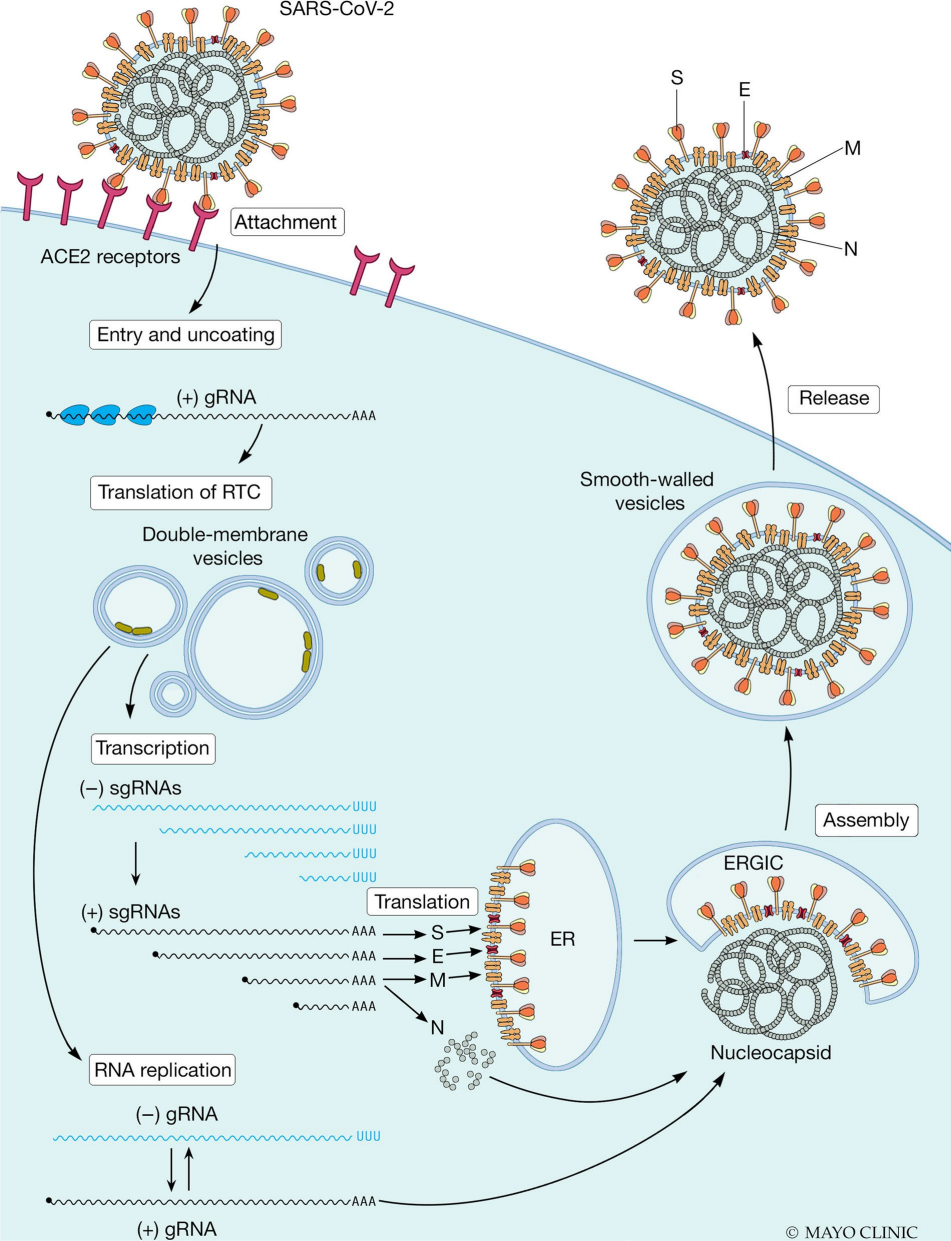
Viral replication pathway of Covid-19. The virus first attaches to the ACE2 receptor and internalizes into the respiratory epithelial cell and causes the release of its genome. The S protein (spikes on the viral surface responsible for attachment to host cell receptors), M protein (shapes the virion, promotes membrane curvature and binds to the nucleocapsid), E protein (helps with viral assembly and release)

Coronaviruses are spherical, positive-sense, single-stranded, non-segmented ribonucleic acid (RNA) surrounded by a lipid capsule derived from the host cell membrane, which has a characteristic surface spike glycoprotein [34]. The general structure comprises of four essential proteins: the spike (S) protein responsible for attachment to host cell receptors, the membrane (M) protein which promotes membrane curvature and binds to the nucleocapsid), the envelop (E) protein which helps with viral assembly and release, and the nucleocapsid N protein (helps with viral replication) [35, 36]. In vitro studies demonstrate that viral non-structural proteins and E2 glycoprotein have a high affinity binding to the porphyrin portion of heme of infected cells [36]. The replication of SARS-CoV-2 is shown in Fig. 1.

SARS-CoV-2 shares structural similarity to both Severe Acute Respiratory Syndrome Coronavirus (SARS-CoV) (approximately 80% similarity) and Middle Eastern Respiratory Disease Coronavirus (MERS-CoV); thereby studies on SARS and MERS are extrapolated and applied to COVID-19 [37]. There is a possibility of emerging strains within SARS-CoV-2, including the so-called S-type and L-type. At present, there is no clear evidence that one strain is more virulent than another, and the two strains do not represent distinct targets for drugs and vaccine development [38, 39].

## SARS-COV-2 invasion of host cell using angiotensin converting enzyme-2 (ACE2) receptor

As shown above, SARS-CoV-2 enters the host by binding to the host cell ACE2 receptor, a membrane-bound protein found on the surface of type 2 pneumocytes, epithelial cells, and enterocytes [34]. In addition to ACE2 mediated endocytosis, direct entry into cells may also occur from cell surface. Transmembrane protease serine 2 (TMPRSS2) is another host cellular protein which has been described as a co-factor for SARS-CoV-2 entry into cells, and Camostat is a clinically relevant drug has been observed to be inhibitory towards SARS-CoV-2 particles in cell culture systems [40].

ACE2 exists in a soluble form in the alveolar fluid where it potentially plays an important role in protection from ARDS [30, 41]. ACE2 receptor expression on epithelial cells increases with age and may partly explain why children are less prone to infection by SARS-CoV-2 [42]. Further, children have higher levels of soluble ACE2 activity within alveolae during ARDS, which has been hypothesized to cause improved lung repair mechanisms compared to adults and be protective against developing COVID-19 [42].

There is controversy regarding the role of ACE inhibitors or ARBs in potentially increasing the virulence of COVID-19 via the upregulation of ACE2 [43]. Although there is currently no convincing evidence to suggest that ACE inhibitors or ARBs have a beneficial or harmful role in COVID-19, further studies are needed to resolve this question. Current consensus by several societies such as the European Society of Cardiology is that there should be no change in the utilization of these agents in patients infected with COVID-19 [44, 45].

Since ACE2 is vital for viral entry to a host cell, a novel treatment strategy utilizing recombinant human ACE2 protein is being studied in a randomized trial in China, looking at its use as a competitive inhibitor as well as a mediator to promote lung repair. Meanwhile, the University of Minnesota has launched a phase 2 clinical trial (NCT04312009) to evaluate the efficacy of losartan, which is an ARB, in COVID-19 pneumonia.

### SARS COV-2 replication and evasion of the immune system

Virus-infected cells typically activate the immune system via cytotoxic cells, interferons or antibodies. The MHC1 (Major Histocompatibility Complex 1) is an antigen presenting cell that causes resultant autophagy by lysosomal degradation. The ORF-2 protein of SARS-CoV-2, which is expressed by both L-type and S-subtypes, down-regulates MHC1 by decreasing total protein and beta-2 microglobulin expression in a dose-dependent and incubation time dependent manner [46]. This causes a decrease in cell surface expression, and hence decreases lysosomal degradation by autophagy, and subsequently decreases cell elimination. This is similar to viruses that cause chronic infection such as HIV (Human Immunodeficiency Virus), which lead to maladaptive immune system while maintaining active replication.

Another mechanism that aids with the elimination of virus-infected cells is increased expression of cytokines. IFN alpha and IFN beta are produced systemically by epithelial cells, monocytes and alveolar macrophages and aggravate lung pathology during respiratory infections. Human intestinal epithelial cells (HIEC) are known to be infected by SARS-CoV-2 and can assist by generating an IFN-mediated intrinsic immune response [47]. IFN gamma is produced locally by lung resident dendritic cells and has been shown to inhibit lung epithelial repair after viral recognition [48, 49]. Non-Structural Protein 1 (NSP1) is another protein that decreases IFN production by preventing translation of IFNs and pro-inflammatory cytokines and IFN-stimulated anti-viral components by binding mRNA translation machinery and enucleated cleavage and degradation of lost mRNA [50].

TLR3 is activated by sensing viral replication from dying cells. It causes increase in IFN gamma, which results in compromise by epithelial cells and predisposes to secondary bacterial infections.

## Emerging anti-viral therapeutic options

Our knowledge of clinical manifestations and pathogenesis of COVID-19 is rapidly evolving as is our understanding of how best to manage this illness and associated complications. Given the rapidly changing data, several therapies are being used solely based on anecdotal evidence. While immediate access to new data is critical in a pandemic such as COVID-19, it can also propagate misinformation as the data is not verified or peer-reviewed in many instances.

Several anti-viral therapies are under investigation for the treatment of COVID-19, either as monotherapies or in combination with other agents [51]. The only medication for which FDA has issued emergency use authorization (EUA) is Remdesivir, which was approved on May 1st, 2020. Table 1 summarizes the key anti-viral therapies currently being investigated in clinical trials. We will discuss some of the promising therapies based on mechanisms of action.

**Table 1:** Emerging anti-viral and immunomodulatory therapies for COVID-19

Drug	Mechanism of action	Adverse effects	Current and planned clinical trials
REMDESIVIR (GS-5734)	Nucleotide/adenosine analog which incorporates into viral RNA causing premature termination of replication Active substrate shown to prevent SARS- and MERS-CoV replication in human airway epithelial cells	Transaminitis Nausea Diarrhea	NCT04257656, NCT04302766, NCT04252664, NCT04292899, NCT04292730
FAVIPIRAVIR (T705)	Synthetic nucleoside analog selectively and potently inhibit the RNA-dependent RNA polymerase (RdRp) of RNA viruses	Nausea and Diarrhea Headache Embryotoxicity	ChiCTR2000030894—Tocilizumab and Faripavir
UMIFENOVIR	Membrane fusion inhibitor developed as a treatment for influenza	Nausea and diarrhea	NCT04260594: monotherapy trial NCT04306497: comparison of various Western medications vs traditional Chinese medications NCT04252885, NCT04273763, NCT04261907, NCT04286503: various combinations
CHLOROQUINE (CQ) and HYDROXYCHLOROQUINE (HCQ)	Endosomal inhibitors: alkalinizes the normally acidic endosomal pH required for virus/cell fusion Decreases glycosylation of receptors which limits viral fusion Presumed to interfere with SARS-CoV binding to hemoglobin Immunomodulatory	Dilated cardiomyopathy Photosensitivity Retinal damage Steven Johnson’s syndrome Should avoid in patients with G-6-PD deficiency or porphyrias	Monotherapy or combination trials: NCT04308668, ChiCTR2000029939, ChiCTR2000029935, ChiCTR2000029899, ChiCTR2000029898, ChiCTR2000029868, ChiCTR2000029837, ChiCTR2000029826, ChiCTR2000029803, ChiCTR2000029762, ChiCTR2000029761, ChiCTR2000029760, ChiCTR2000029741, ChiCTR2000029740, ChiCTR2000029609, ChiCTR2000029559, ChiCTR2000029542
HCQ + AZITHROMYCIN	Azithromycin prevents bacterial superinfection	Prolonged QTC Arrythmias (concern given cardiomyopathy associated with COVID-19)	NCT04322123, EU Clinical trials register number 2020-000890-25
GELDANAMYCIN	Heat shock protein inhibitor; degrades endosomal activity, thereby interferes with viral replication in host cells	Hepatotoxicity Anemia	No registered trials on *clinicaltrials.gov* yet Has antitumor activity by inducing cell apoptosis
LOPINAVIR (LPV) AND RITONAVIR (RTV) ± RIBAVIRIN	Lopinavir: Protease inhibitor; inhibits assembly of mature virions Ritonavir: CYP3A4 inhibitor; slows the metabolism of lopinavir Ribavirin: inhibits IMP dehydrogenase. Some evidence that Ribavarin targets a protein that SARS-Co-V-2 directly binds to	Due to CYP3A4 interaction, can cause significant drug-drug interaction GI disturbances (diarrhea, nausea, and vomiting) Lipid abnormalities	NCT04307693, NCT04255017, NCT04261907, NCT04276688, NCT04303299, NCT04303299 MIRACLE trial, NCT02845843 Initial studies from China showing no change in survival or recovery speed, although high mortality rate suggests patient population was only severely ill
COMBINATION ANTIVIRAL THERAPY	Remdesivir Lopinavir/Ritonavir (Kaletra/Aluvia) Chloroquine Lopinavir/Ritonavir & IFN beta	Due to CYP3A4 interaction, can cause significant drug-drug interaction GI disturbances (diarrhea, nausea, and vomiting) Lipid abnormalities	- NCT04321616: SOLIDARITY trial currently in Argentina, Bahrain, Canada, France, Iran, Norway, South Africa, Spain, Switzerland, and Thailand). Endpoints: mortality rate, length of hospitalization, utilization of ICU and ventilators
DARUNAVIR AND COBICISTAT	HIV Protease Inhibitors	Diarrhea Nausea Vomiting Headache	NCT04252274, NCT04303299 ChiCTR2000029541: Prezcobix vs. lopinavir-ritonavir combination combined with thymosin a1
OSELTAMIVIR	viral neuraminidase inhibitor (enzyme found on the surface of the influenza virus)	Transaminitis Nausea Diarrhea	NCT04261270: Monotherapy: NCT04303299: Combination therapy
DANOPREVIR (RG7227/ITMN-191) & RITONAVIR & INTERFERON	Danoprevir: Non-covalent macrocyclic inhibitor of HCV NS3/4A protease Ritonavir: CYP3A4 inhibitor; slows the metabolism of lopinavir Interferon: cytokine	Nausea Anorexia Transaminitis	NCT04291729
LERONLIMAB (PRO 140)	Humanized IgG4 monoclonal antibody CCR5 antagonist	Diarrhea Headache Lymphadenopathy Hypertension	No registered trial on *clinicaltrials.gov* yet Phase 2 clinical trial planned Currently being studied as combination therapy with HAART for HIV-infected patients, and for metastatic triple-negative breast cancer
APN01 (RHACE2)	Recombinant human angiotensin-converting enzyme 2	Awaiting study results	NCT04287686: Pilot trial
LOSARTAN	Angiotensin receptor blocker; blocks viral entry	Kidney injury Hypotension	NCT04312009, NCT04311177
CORTICOSTEROIDS	Presumed to decrease inflammation and hence pulmonary fibrosis Was recommended only if alternative indication exists, e.g. refractory ARDS, sepsis or septic shock Dexamethasone recently recommended for disease requiring oxygen supplementation	Hyperglycemia Psychosis Secondary infection Avascular necrosis	NCT0424459, ChiCTR2000029656, ChiCTR2000029386, NCT04323592, NCT04244591 Used in severe disease Different preparations, doses and schedules being studied
THALIDOMIDE	Blocks NF- ƙB binding to gene promotors, reducing the production of IL-6, TNF-α and chemokines	Well known teratogen	NCT04273529, NCT04273581
TOCILIZUMAB	IL-6 receptor antibody	Neutropenia Transaminitis Immunosuppression	NCT04317092: TOCIVID-19 with use in COVID pneumonia ChiCTR2000030894—Tocilizumab and Faripavir ChiCTR2000029765)—Tocilizumab alone
SARILUMAB (REGN88)	IL-6 receptor antagonist	Neutropenia Transaminitis Nasal congestion	NCT04315298, NCT04327388 and NCT04324073: CORIMUNO-19-SARI trial A trial planned with combination with remdesivir
SILTUXIMAB	Chimeric anti-IL-6 monoclonal antibody Binds to soluble and membrane-bound forms of IL-6	Cytopenias Edema Hypotension Increased risk of secondary infections Hyperuricemia	No registered trials on *clincialtrials.gov*
ANAKINRA	IL 1 inhibitor	Hypersensitivity Neutropenia Immunosuppression	Currently being evaluated for neurotoxicity in CAR-T neurotoxicity: NCT04150913 and active rheumatoid arthritis: NCT00117091 Planned but no registered trial for COVID-19 yet on *clinicaltrials.gov*
RUXOLITINIB	JAK-STAT inhibitor	Pancytopenia Orthostatic dizziness Vertigo Hyperlipidemia	ChiCTR 2000029580: MSC + ruxolitinib versus ruxolitinib alone
FEDRATINIB (SAR302503, TG101348)	Selective JAK2 inhibitor Decreases the IL-17 production and IL-22 production by Th17 cells Inhibits GM-CSF communication via the JAK pathway	Pancytopenia Hyperlipidemia	Preclinical data by Wu et al*.* Journal of Microbiology, Immunology and Infection. Online March 11, 2020 No registered trials on *clincialtrials.gov*
BARICITINIB	JAK inhibitor	Drowsiness Visual problems	NCT04320277: BARI-COVID study of baricitinib in symptomatic patients
RECOMBINANT INTERFERON THERAPY	Stimulates immune response to inhibit viral replication	Hypersensitivity reactions Immunosuppression	NCT04320238: Inhaled recombinant human interferon alpha-1b NCT04315948, NCT04276688: IFN infusion in combination with LPV/RTV
CAMRELIZUMAB	Anti-PD-1 antibody	Hypersensitivity Neutropenia Immunosuppression	NCT04268537: Camrelizumab and Thymosin combination
ECULIZUMAB	Distal complement inhibitor	Risk of severe meningococcal and pneumococcus infections, requiring vaccinations before initiating therapy	NCT04288713: SOLID-C19 study
NATURAL KILLER (NK) CELL THERAPY	Cytotoxic to virally infected cells Non-MHC dependent cytotoxicity Secrete cytokines to generate a potent anti-viral immune response	Infusion reactions	NCT04280224: NK cell therapy in COVID-19 associated pneumonia
MESENCHYMAL STEM CELL (MSC) THERAPY	Cell therapy with regenerative properties potentially can prevention of lung fibrosis in COVID-19 associated lung injury	Infusions may cause microcirculatory blockage, aerosolized MSC-exosome does not carry this risk	ChiCTR 2000029580: phase 1 clinical trial in combination with ruxolitinib versus ruxolitinib alone NCT04252118, NCT04288102, NCT04273646, NCT04269525: Uses umbilical cord derived MSC NCT04276987: Uses MSC exosomes in COVID-19 Different sources, preparations, doses and schedules of MSC in different studies
CONVALESCENT PLASMA THERAPY	Treatment aimed at isolating and transfusing protective antibodies from plasma of recovered patients	Transfusion reactions	NCT04321421: COV-19-PLASMA study in critically ill patients NCT04325672

### Viral mRNA synthesis inhibitors

Remdesivir (GS-5734) is a viral nucleotide (adenosine) analog, which incorporates into nascent viral RNA chains and results in premature termination [51–53]. In vitro studies suggest that the combination of remdesivir and CQ could inhibit viral replication even at low concentrations [51]. The multi-center trial, commonly known as SOLIDARITY (NCT04321616), was one of the first large-scale trials aiming to compare HCQ, remdesivir, and the combination of HCQ/remdesivir in hospitalized COVID-19 patients. This trial was started as a five-arm adaptive design trial aimed at studying the primary outcome of in-house mortality at 3 weeks. Other outcomes measures included comparison of mechanical ventilation occurrence, viral clearance, and markers of inflammation [54]. On March 24th, 2020, the Czech Republic approved the use of remdesivir in critically ill patients [39]. Some adverse effects of remdesivir are transaminitis, nausea, and vomiting—further studies are underway to evaluate for side effects.

Based on the finding of the Adaptive COVID-19 treatment trial (ACTT), Remdesivir may be most beneficial if given to patients with severe COVID-19 lung involvement before mechanical ventilation [55]. Remdesivir use was associated with a reduced median time to recovery (11d v 15 days). A mortality benefit was observed (8% v 11.6%) but was not statistically significant.

Favipiravir (T-705), a synthetic nucleoside analog, functions as a chain terminator at the site of incorporation of the viral RNA, thereby inhibiting the RNA-dependent RNA polymerase. Umifenovir is a membrane fusion inhibitor originally developed for influenza. A multicenter trial comparing favipiravir to umifenovir in COVID-19 patients with moderate symptoms and chest imaging abnormalities showed a 71% recovery at day 7 for those receiving favipiravir, compared to 55% in the group receiving umifenovir [43]. Another trial in China on 80 patients infected with SARS-CoV-2 compared response to Interferon (IFN)-α combination with either favipiravir or lopinavir (LPV)/ritonavir (RTV). The combination with favipiravir was shown to expedite viral clearance (4 days versus 11 days, shorten recovery time (statistically significant at 92% versus 62%) and cause improvement in chest imaging [56]. Based on these studies, Favipiravir received marketing approval in China on February 17th, 2020. Common side effects of favipiravir include nausea, headache, and diarrhea. Favipiravir may cause embryotoxicity [57].

### Endosomal function inhibitors

CQ and its derivative, HCQ have historically been used for malaria and amebiasis. They are also widely utilized in the treatment of auto-immune conditions such as rheumatoid arthritis (RA) and systemic lupus erythematosus (SLE), and in recent years have been seen to have activity against a wide range of viruses in-vitro such as Ebola virus and SARS-CoV-1 [58, 59].

Their anti-viral effects are achieved by multiple mechanisms, primarily by alkalinizing the normally acidic endosomal pH of the infected cells, limiting virus-cell fusion, and modifying glycosylation of receptors [60]. They also affect cell signaling and have an immunomodulatory effect by blocking proinflammatory cytokines, particularly tumor necrosis factor-alpha (TNFα) and IL-6, IL-1β production by activated alveolar macrophages and downregulation of TNF receptors on monocytes resulting in decreased monocyte activation. They also reduce the severity of ARDS by decreasing TNF-α mediated opening of tight junctions of epithelial cells, as well as upregulation of leukocyte adhesion molecules (LAM) and hence decreasing leukocyte extravasation into damaged alveoli [61, 62]. Apart from these, another mechanism by which CQ/HCQ is postulated to interfere with SARS-CoV-2 relates to the virus’s ability to block hemoglobin synthesis. CQ/HCQ competes with the porphyrin to bind to the E2 portion of the virus, thus freeing the porphyrin to incorporate into hemoglobin [36].

Based on these observations, these agents were thought to be promising prophylactic and therapeutic options for COVID-19. HCQ was investigated both as monotherapy as well as in combination with Azithromycin (an antibiotic added to prevent bacterial super-infection) for COVID-19 [63]. Preliminary data from the open-label non-randomized French trial on six patients of this combination showed viral load reduction [63]. On March 28, 2020, Chloroquine and Hydroxychloroquine sulfate were issued EUA to treat hospitalized adults and adolescents who were unable to be enrolled in a clinical trial. However, based on continuous review of the available scientific evidence, the EUA was revoked on June 15, 2020, as there was no clear demonstratable effect [64].

Adverse effects of CQ and HCQ include prolonged QTc interval, arrhythmias, and dilated cardiomyopathy which may be of concern given the high incidence of cardiac arrhythmias and sudden cardiac death being noted in critically ill COVID patients [65–67]. Photosensitivity and retinal damage can also occur, and rare cases of Stevens-Johnson syndrome have been reported [68]. They should be avoided in patients with glucose-6-phosphate-dehydrogenase (G-6-PD) deficiency or porphyrias [67].

### Other therapies to inhibit viral host attachment and viral replication

LPV and RTV are both antiviral agents used in combination to treat human immunodeficiency virus (HIV). LPV is a protease inhibitor that acts by preventing maturation of the HIV-1 virus, while RTV is a cytochrome P3A4 (CYP3A4) inhibitor which increases plasma levels of LPV. In a randomized study, involving 199 patients with severe COVID-19, there was some anecdotal success, but the combination did not demonstrate significant improvement in death rates, rates of oxygen desaturation, and rates of intubation compared to the standard of care treatment in matched controls [36]. In SARS, LPV and RTV were most effective when used in combination with ribavirin [69]. There is some evidence based on viral-host cell proteomic mapping demonstrating SARs-CoV-2 binds directly to a protein that is targeted by ribavirin [70]. Adverse effects may include diarrhea, nausea, rash, and asthenia.

Other anti-viral therapies currently in clinical trials are summarized in Table 1.

## COVID associated cytokine storm and lung injury

Some patients with COVID-19 undergo progressive clinical decline which is characterized by the following phases: early phase, pulmonary phase, and hyper inflammation phase. We describe the phases of COVID-19 infection, discuss immune dysregulation resulting in cytokine storm, and review immunomodulatory options being studied in this disease. Therapeutic options are summarized in Table 1.

### Early phase: phase one

The first phase of COVID-19 infection generally presents as fever and cough triggered by robust viral replication within the respiratory epithelium. The innate immune system is the primary mediator of inflammation during this phase. Viral particles are recognized by Toll-like receptors (TLR) on macrophages, neutrophils and dendritic cells (DC), which typically activate nuclear factor kappa-light-chain-enhancer of activated B cells (NF-*ƙ*β) pathways leading to transcription of several cytokines including IL-6 and IFNƴ [71–73]. IFNs induce Janus Kinase (JAK) and activator of transcriptor (STAT) pathways, which promote expression IFN-stimulation genes [74]. SARS-CoV-2 appears to inhibit the NF-*ƙ*β -TLR4 pathway and thereby delays IFN production, allowing unchecked viral replication [75]. The N protein of SARS-CoV-2 appears to antagonize the host response and the IFN response. This provides a potential therapeutic target, as utilizing IFN gamma earlier could help modulate the immune response and potentially decrease disease severity.

### Pulmonary phase: phase two

Progression to the second phase of SARS-CoV-2 infection is characterized by the development of hypoxia. The direct virus-induced cytopathic effect on type two pneumocytes via ACE2 receptors activates the innate immune system, resulting in a large influx of monocytes, macrophages, and heavy infiltration of neutrophils [76]. They produce nitric oxide, growth factors, and transforming growth factor-beta (TGF-β) which contribute to oxidative injury, leading to capillary leak and alveolar basement membrane damage utilizing the TLR-4- NF-*ƙ*β pathway [77]. Loss of the type two pneumocytes also impairs endogenous lung repair mechanisms, leading to the cascading progression of injury.

### Hyperinflammation phase: phase three

As COVID-19 disease progresses to Phase 3, which appears approximately 9–12 days after the onset of illness, there may be development of ARDS, CRS, septic shock and cardiac complications [9, 18].

SARS-CoV-2 drives a lower anti-viral transcriptional response compared to other respiratory viruses, resulting in low IFN-I and IFN-III levels and elevated chemokine levels [78]. T lymphocytes, activated macrophages, and neutrophils migrate towards and infiltrate the alveolar microenvironment, releasing pro-inflammatory chemokines and cytokines including IL-1, IL-6, and IL-8, IL-17 and TNF [79, 80].

**Fig. 2:**
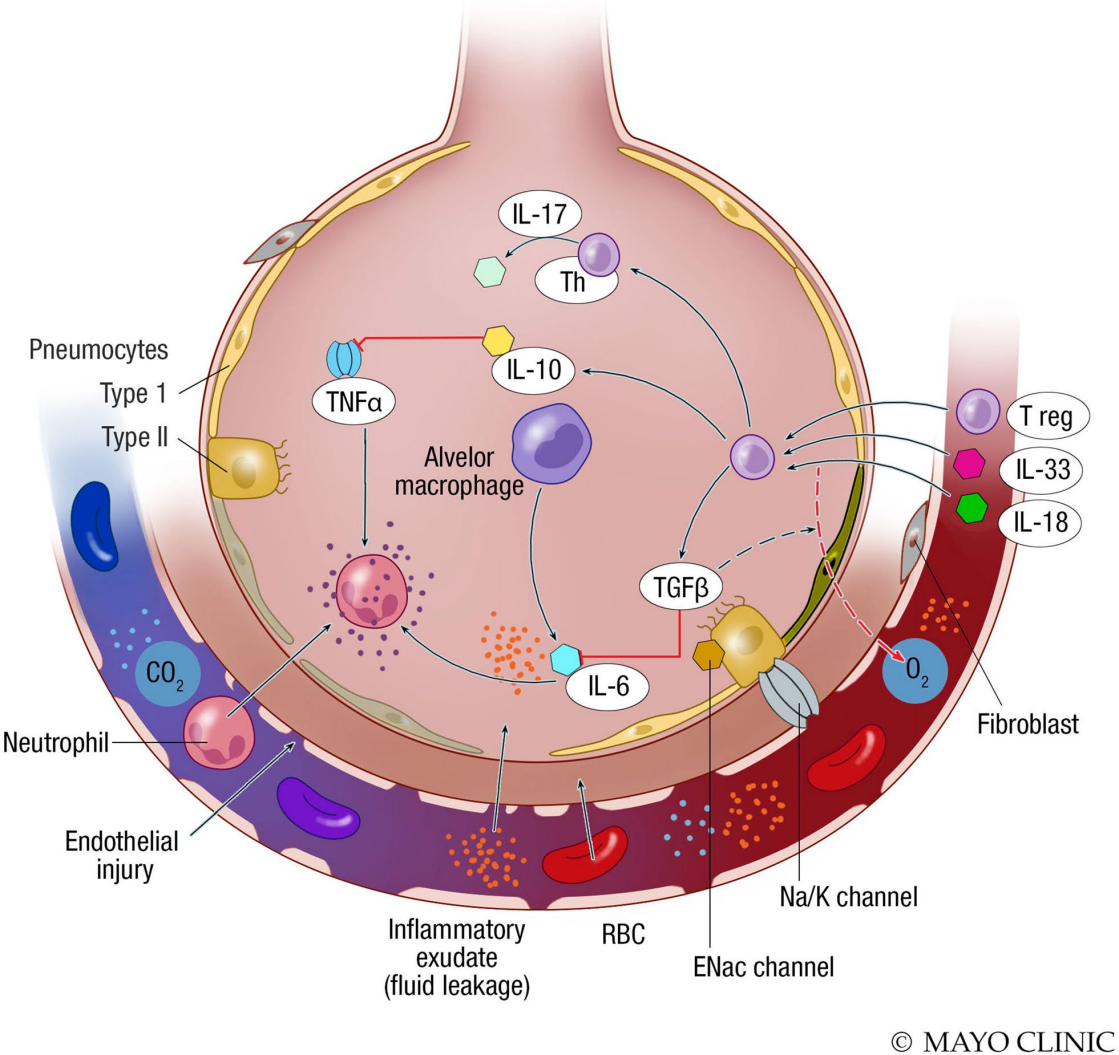
Alveolar micro-environment showing pathophysiology of acute respiratory distress syndrome (ARDS). The Th response causes the release of IL-17 which activates TNF alpha which enhances epithelial injury and activates neutrophils to cause degranulation. IL-6 is produced by alveolar macrophages which also stimulates neutrophils. Once the epithelial integrity of alveolus is breached epithelial sodium channel (Enac) channels and Na/K channels fail to maintain homeostasis eventually leading to an increase in permeability of capillaries causing exudation of fluids. T reg cells also trigger TGF beta which causes fibrosis to the damaged epithelial membrane. Most of the COVID-19 patients present with ground-glass opacities and fibrosis of their lungs

Cytokines implicated in COVID-19-associated lung injury and CRS include IL-1β, TNF-α and IL-6, which activate other proinflammatory pathways via the JAK-STAT pathway and activation of Th cells [17, 77, 81, 82]. IL-6 also recruits macrophages at sites of injury and promotes inflammation and ARDS. The SARS E protein causes IL-1β secretion, leading to lung inflammation and injury [83]. IL-1β also accelerates the production of TNF-α, which in turn promotes the apoptosis of lung epithelial and endothelial cells [84]. IL-1β, TNF-α and IL-6 provide an integrated, amplified inflammatory response, leading to a breach in alveolar basement membrane integrity. This subsequently increases vascular permeability, leading to pulmonary edema, which is associated with pulmonary deterioration even with ventilator support. This is schematically represented in Fig. 2.

Secondary hemophagocytic syndrome (sHLH) may also develop during this phase, and is characterized by fever, cytopenias, hyperferritinemia, and an increase in proinflammatory cytokines including IL-6 and IL-18 [85, 86]. This could be useful to consider when treating a patient with COVID-19—white blood cell count, CRP, and D-dimer may be helpful to monitor and to distinguish between CRS versus secondary bacterial infections.

Another aspect that warrants further study is the consequence of elevated D-dimer, as it is unclear at present if it is reflective of an acute phase only, or potentially a disseminated intravascular coagulopathy (DIC) phenomena occurring in the lungs secondary to the sHLH.

**Fig. 3:**
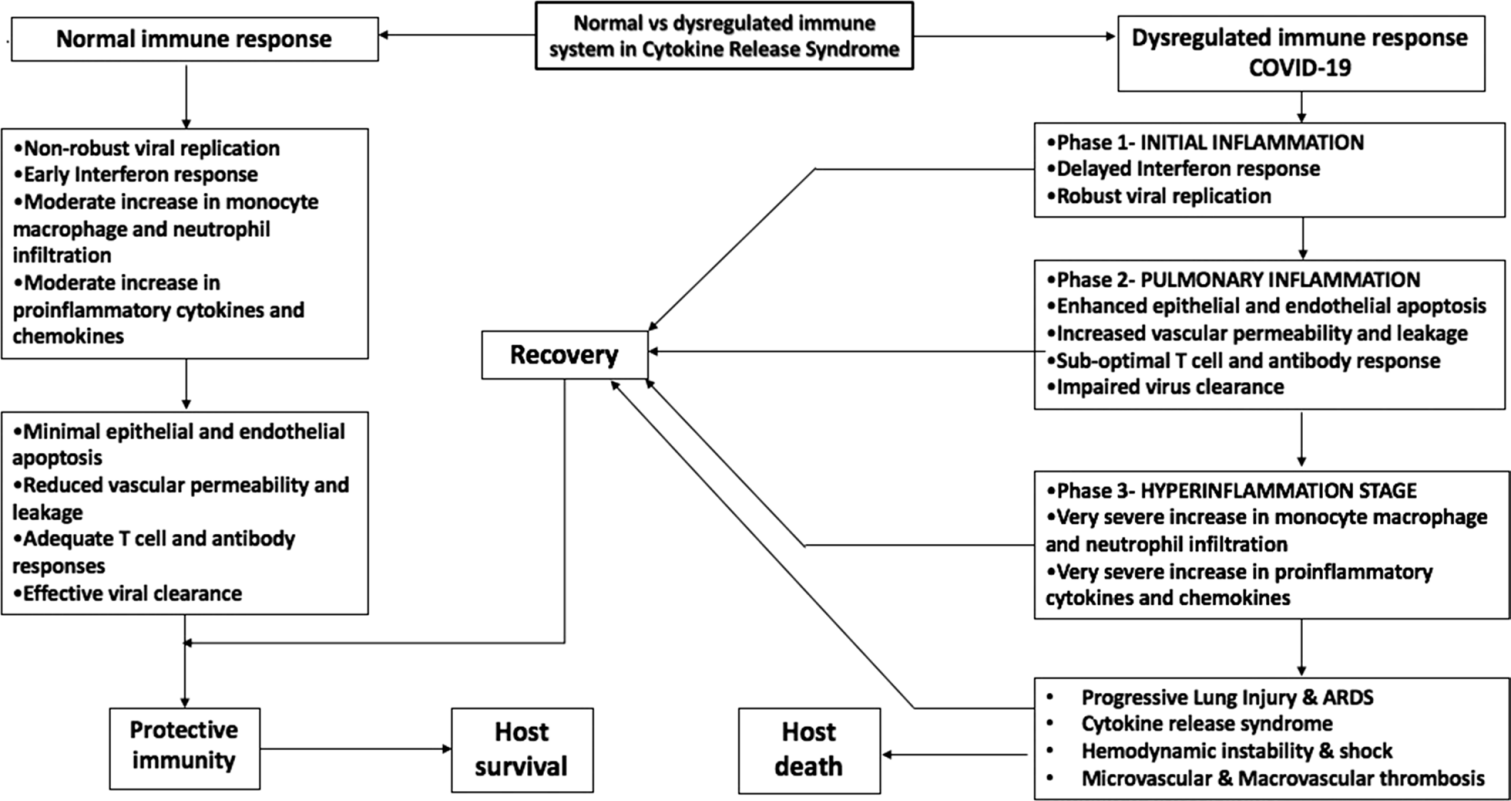
Immune dysregulation in cytokine release syndrome. The involvement of the immune system in COVID-19 is in 3 phases: initial inflammation which is characterized by delayed interferon response and robust viral replication, the pulmonary inflammation phase which is characterized by sub-optimal T-cell and antibody response, leading to increased vascular leakage and permeability and impaired viral clearance, and the hyperinflammation phase which is characterized by very severe infiltration of monocytes, macrophages and neutrophils—this leads to progressive lung injury and ARDS as well as hemodynamic instability and shock

An overview of the immune dysregulation in COVID-19 is shown in Fig. 3.

### Recovery phase

This phase can occur at any time during the disease and is divided into the early recovery stage (ERS) and the late recovery stage (LRS). IL-6 production and circulating inflammatory monocytes were noted in ERS, with the potential for ongoing lung injury. In LRS, patients’ serum contains an abundance of antibodies. It is speculated that dendritic cells (DC) produce IL-18 which promotes proliferation of B cells as well as IL-7 which promotes T cell proliferation, IL-2 secretion and B cell proliferation, and antibody production [87].

## Non-selective immunomodulators

### Corticosteroids

The use of corticosteroids was much debated for COVID-19 as steroids are immunosuppressive, potentially delaying viral clearance and increased risk of secondary infection. An observational study on intensive care unit (ICU) patients with MERS reported that high doses of corticosteroids were associated with more severe disease but did not increase ninety-day mortality [88]. Other meta-analyses in SARS showed no benefit of steroid use [89, 90]. An observational trial by Yuan et al. showed no benefit from Methylprednisone. However, preliminary data from the UK RECOVERY trial showed that low dose dexamethasone (6 mg PO or IV daily for 10 days) reduced mortality by 35% in intubated patients and by 20% in hospitalized patients requiring oxygen supplementation compared to patients receiving standard of care, but had no effect in patients who did not require oxygen supplementation [91]. Based on these data, Dexamethasone is now recommended for patients with severe COVID-19 (requiring oxygen) including those on mechanical ventilation by the NIH and IDSA [92, 93].

#### Non-steroidal anti-inflammatory drugs (NSAIDs)

There is controversy regarding NSAID use for symptom relief with COVID-19. The French National Agency for Medicines and Health Products Safety suggested that COVID-19 viral clearance may be delayed by NSAIDs. The European Medical Association (EMA) did not support this statement, due to lack of supporting evidence [94].

#### Thalidomide

Thalidomide blocks the NF-*ƙ*B binding to gene promotors, reducing the production of IL-6, TNF-α and chemokines [95]. It increases circulating NK cells and increases IFN-ƴ production by T cells. It is FDA approved to treat multiple myeloma in combination with low dose dexamethasone and trials suggest activity in influenza-associated lung injury. Trials are evaluating the role of Thalidomide in COVID-19 (NCT04273529). This drug has well-known teratogenic effects [96].

### Cytokine inhibitors

#### IL-6 inhibitors

IL-6 is one of the key players in accelerating a cytokine storm. Several IL-6 antagonists are being studied for safety and efficacy in COVID-19. Tocilizumab, a prototype IL-6 receptor antagonist, is the most studied and is currently used in managing cytokine storm in chimeric antigen receptor Antibody (CAR-T) therapy. It was approved in China for the treatment of severe COVID-19 in March 2020. An initial trial in China where Tocilizumab was administered to 20 patients with severe COVID-19, targeting cytokine storm demonstrated promising results, with 19 patients discharged in stable condition 2 weeks after administration. Chest imaging showed significant improvement on day four to five [97]. Anecdotal reports from other large centers suggest rapid improvement in some patients with improved oxygenation often within 24 to 48 h of administration. Also, treatment may be more effective earlier in the disease course than when ARDS fully develops. Typically, a single 8 mg/kg dose is administered. Notable adverse effects of Tocilizumab include increased risk of secondary infection, liver dysfunction, and cytopenias [98].

A similar anti-IL-6 agent, Sarilumab, is being investigated in clinical trials for COVID-19 (*e.g*. NCT04315298). Siltuximab is a chimeric anti-IL-6 monoclonal antibody that binds to soluble and membrane-bound forms of IL-6, preventing binding to soluble and membrane-bound receptors. It is used in the treatment of CAR-T induced CRS not responding to tocilizumab, and hence may play a role in COVID-19 induced CRS as well [99, 100]. Side effects include cytopenias, edema, hypotension, and increased risk of secondary infections.

#### IL-1 inhibitors

IL-1 is another pro-inflammatory cytokine that feeds the cytokine storm. It mediates inflammation in the lungs, leading to fever, ARDS and fibrosis. Anakinra, an IL-1 blocker, is used to treat RA in adults and neonatal-onset multisystem dysfunction (NOMID), as well as used off-label for neurotoxicity complications of CAR-T therapy. This is currently being investigated for COVID-19 induced CRS [85, 101]. Adverse effects include hypersensitivity, neutropenia, and infections [102].

### JAK inhibitors

The regulation of the JAK-STAT pathway is essential for cross interaction between various cytokine signaling pathways leading to an uncontrolled pro-inflammatory state. JAK inhibitors such as Ruxolitinib and Fedratinib target the pro-inflammatory JAK/STAT pathway and are approved for myeloproliferative disorders [17, 103, 104]. Ruxolitinib is also approved for steroid-refractory graft versus host disease (GVHD) which are JAK/STAT-driven diseases. Baricitinib is another JAK inhibitor currently used in RA which is being investigated for efficacy in COVID-19 (NCT04320277). It also may have anti-viral activity by reducing clathrin-mediated endocytosis [105]. Common dose-limiting side effects of JAK inhibitors include cytopenias, hyperlipidemia and increased risk of secondary infection.

### Immune effector cell therapy

#### NK cell therapy

NK cells are recruited to site of infection by chemokines, activated by cytokines produced from infected cells, like IL-12, IL-15, IL-18, and IFN. Activated NK cells counter the virus by increased IFN-ƴ production and NK cell-mediated cytolysis of infected cells. Possible blunting of NK responses by SARS-CoV-2 may allow disease progression [21, 106, 107]. Given its anti-viral properties, allogeneic, “off the shelf”, NK cell infusions, derived from healthy donors, are being evaluated for efficacy in COVID-19 associated pneumonia. NK infusions are generally well tolerated.

#### Mesenchymal cells (MSC)

Cell-based therapy, especially mesenchymal stem cell therapy, is considered to be one of the most promising therapeutic approaches aiming to provide opportunities to treat several diseases. MSC have diverse immunomodulatory and regenerative properties [54]. Previous trials have shown evidence of stabilized and improved lung function in patients with ARDS who received MSC without any treatment-related adverse effects. Given the hypothesis that MSC therapy might prevent the triggering of cytokine storm and promote endogenous repair, several clinical trials are Looking at the safety and therapeutic potential of MSC from various sources in SARS-CoV-2 (e.g. NCT04313322) [108, 109]. Since infusions may carry the risk of microcirculation injury, MSC derived exosomes, which can be delivered by aerosol inhalation, are also being evaluated for safety and efficacy in severe COVID-19 pneumonia (NCT04276987). Availability and large-scale manufacturing are potential issues.

### Complement inhibitors

In addition to DIC, the complement pathway contributes to lung injury in SARS, and it may contribute to the high incidence of fatal microvascular and macrovascular thrombosis associated with COVID-19 [110]. Eculizumab, which is approved to treat rare complement-mediated disorders like paroxysmal nocturnal hemoglobinuria (PNH), atypical hemolytic uremic syndrome (aHUS), neuromyelitis optica spectrum disorder and myasthenia gravis, is being evaluated for safety and efficacy in COVID-19 (NCT04288713). Immunosuppression is a major side effect. In general, patients should be vaccinated against meningococcus and pneumococcus prior to use [111]. However, this may not be possible in COVID-19 patients being considered for this therapy.

### Programmed cell death (PD)-1 inhibitors

These likely function by delaying T cell exhaustion. Camrelizumab, a fully humanized PD-1 monoclonal antibody, is currently approved to treat lymphoma in China and is now being investigated as an immunoregulatory therapeutic option for COVID-19. Clinical efficacy of camrelizumab plus thymosin in patients with COVID-19 will be evaluated in clinical trial NCT04268537 [112]. Adverse effects of immunotherapy are generally related to breakthrough autoimmunity and may include rash, diarrhea, colitis, and thyroid dysfunction [112].

### Therapies utilizing passive immunity

Convalescent plasma exchange, which utilizes passive immunity, may be an effective treatment strategy. Serum rich in anti-SARS-CoV-2 Ab can be obtained from recovered donors and transfused to infected patients. Shen et al*.* reported transfusing hyperimmune plasma on 5 critically ill patients infected with COVID-19, who had severe pneumonia, rapid progression, and persistently high viral load despite treatment, as well as severe ARDS mechanical ventilation. These patients received transfusion with convalescent plasma with SARS-CoV-2v-2 specific antibody with the resultant resolution of ARDS within two weeks, and 3 of these patients were extubated within 2 weeks. All patients clinically improved around a week later [113]. This appears promising and is being investigated in several countries for critically ill patients [114].

A summary of all the therapies discussed above is listed in Table 1.

## Conclusion

Given the worsening trajectory of the COVID-19 pandemic, there is a global race to develop effective therapeutic interventions. Since SARS-CoV-2 is a novel virus, our understanding regarding its host interaction and resultant inflammatory responses is still evolving. Most therapeutic agents currently under investigation are based on prior observations with SARS or experience in immune dysregulation. Moreover, with rapid publication pace and immediate access to data before formal peer-review, there are emerging challenges in ensuring the accuracy of published information for clinical use. The first medication to receive EUA was HCQ/chloroquine. However, based on subsequent data, this EUA was revoked. Currently, the only direct anti-viral agent with EUA for COVID-19 is Remdesivir.

A key mechanism driving COVID-19 associated mortality may be the cytokine storm augmenting lung injury. While the precise pathways driving CRS and ARDS are yet to fully understood, high levels of pro-inflammatory cytokines such as IL-6, IL-1β, and TNF-α characterize the cytokine storm. There is encouraging preliminary data in CRS and ARDS with the immunomodulators like Tocilizumab, an IL-6 inhibitor. These agents may be used alone or in conjunction with other treatments, such as dexamethasone, in severe disease.

Cellular therapy may also have a role in treating and reducing lung injury in COVID-19. Based on their application as cancer treatments, NK cells are known to exert direct cytotoxic effects on virally infected cells and produce IFN-ƴ and TNFα to boost the host immune response. MSCs, with prior use in the treatment of GVHD, fibrotic liver, and lung diseases, may also improve COVID-19 associated lung damage. There is a potential role for the development of agents aimed at enhancing immune surveillance by specifically targeting ORF8 or NSP1 to impair MHC1 antigen presentation.

Convalescent plasma from recovered patients is also an attractive treatment option for critically ill or rapidly deteriorating patients. Ultimately, the hope is to develop vaccinations effective in prevention, but this may take several months or years to develop.

Most of the evidence on current therapeutic agents are based on small observational studies and need to be validated by larger studies and RCTs. Given the paucity of information regarding therapeutic agents and their administration, there is an urgent need for studies to evaluate all aspects of therapy, including the timing of administration, potential synergism between treatments, and potential toxicities. It is also crucial to balance the need to expedite the utilization of potentially helpful medications with the need to ensure patient safety.

## Data Availability

Not applicable.

## Abbreviations

SARS-CoV-2: Severe Acute Respiratory Syndrome Coronavirus 2
COVID-19: Coronavirus disease 2019
ARDS: Adult respiratory distress syndrome
CRS: Cytokine release syndrome
IL: Interleukin
WHO: World Health organization
CDC: Centers for disease control
CSSE: Center for Systems Science and Engineering
ACE2: Angiotensin converting enzyme
TLR: Toll like receptors
NF-ƙB: Nuclear factor kappa-light-chain-enhancer of activated B cells
IFN: Interferon
TNF: Tumor necrosis factor
SARS: Severe Acute Respiratory Syndrome
MERS: Middle East Respiratory Syndrome
USA: United States of America
pro-BNP: Pro brain-type natriuretic peptide
CRP: C-reactive protein
T cells: T lymphocytes
B cells: B lymphocytes
NK: Natural killer
β-CoV: Beta coronavirus
SARS-CoV: Severe Acute Respiratory Syndrome Coronavirus
MERS-CoV: Middle Eastern Respiratory Disease coronavirus
rt RT-PCR: Real time reverse transcription polymerase chain reaction
BAL: Bronchoalveolar lavage
CT: Computed tomography
mRNA: Messenger ribonucleic acid
NSP1: Nonstructural Protein 1
TMPRSS2: Transmembrane protease, serine 2
ARB: Angiotensin II type I receptor blockers
NHC: National Health Commission
CQ: Chloroquine phosphate
HCQ: Hydroxychloroquine
IFN: Interferon
LPV: Lopinavir
RTV: Ritonavir
RA: Rheumatoid arthritis
SLE: Systemic lupus erythematosus
E: Envelop protein
LAM: Leukocyte adhesion molecules
G6PD: Glucose-6-phosphate-dehydrogenase
CYP3A4: Cytochrome P450 3A4
HIV: Human immunodeficiency virus
JAK: Janus kinase
STAT: Activator of transcriptor pathway
N protein: Nucleocapsid protein
M: Membrane protein
S: Spike protein
sHLH: Secondary hemophagocytic syndrome
ERS: Early recovery stage
LRS: Late recovery stage
DC: Dendritic cells
NSAIDs: Non-steroidal anti-inflammatory drugs
EMA: European Medical Association
FDA: U.S. Food and Drug Administration
CAR-T: Chimeric antigen receptor T-cells
MSC: Mesenchymal cells
PNH: Paroxysmal nocturnal hemoglobinuria
aHUS: Atypical hemolytic uremic syndrome
PD-1: Programmed cell death-1
Ig: Immunoglobulins
DC: Dendritic cells
ICU: Intensive care unit
COPD: Chronic obstructive pulmonary disease
NOMID: Neonatal onset multisystem dysfunction
